# Angiotensin-Inhibiting Drugs Do Not Impact Disease Activity in Patients with Rheumatoid Arthritis: A Retrospective Cross-Sectional Study

**DOI:** 10.3390/jcm10091985

**Published:** 2021-05-05

**Authors:** Dorien M. C. F. Sluijsmans, Daphne C. Rohrich, Calin D. Popa, Bart J. F. van den Bemt

**Affiliations:** 1 Department of Rheumatology, Sint Maartenskliniek, Hengstdal nr 3, 6574 NA Ubbergen, The Netherlands; doriensluijsmans@gmail.com (D.M.C.F.S.); d.rohrich@maartenskliniek.nl (D.C.R.); 2 Department of Pharmacy, Sint Maartenskliniek, 6574 NA Ubbergen, The Netherlands; b.vandenbemt@maartenskliniek.nl; 3 Department of Rheumatology, Radboud University Nijmegen Medical Centre, 6525 GA Nijmegen, The Netherlands; 4 Department of Pharmacy, Radboud University Medical Centre, 6525 GA Nijmegen, The Netherlands

**Keywords:** rheumatoid arthritis, cardiovascular, ACE inhibitors, angiotensin II receptor blockers

## Abstract

Objectives: Besides their proven effectivity in decreasing the risk of cardiovascular events, angiotensin-converting enzyme inhibitors (ACEi) and angiotensin II type 1 receptor blockers (ARBs) are likely to possess anti-inflammatory properties as well. This study aims to investigate whether the use of ACEi and ARBs additionally lowers disease activity in patients with rheumatoid arthritis (RA). Methods: In this cross-sectional study, we used ARBs or ACEi to study RA patients who had at least one DAS28-CRP measurement during a one-year period. A control group of RA patients without ACEi/ARBs was randomly selected. The primary outcome was the difference between the DAS28-CRP scores of ACEi/ARBs users and controls. The secondary outcomes were the differences between administered dosages of csDMARDs and bDMARDs for users and controls, respectively; these were expressed in defined daily dose (DDD). Confounders were included in the multiple regression analyses. Results: A total of 584 ACEi/ARBs users and 552 controls were finally examined. Multiple linear regression analyses showed no association between the use of ACEi or ARBs and the DAS28-CRP scores (ACEi factor 1.00, 95% CI 0.94–1.06; ARBs 1.02, 95% CI 0.96–1.09), nor with the dosage of csDMARDs (ACEi 0.97, 95% CI 0.89–1.07; ARBs 0.99, 95% CI 0.90–1.10). Furthermore, the use of ACEi was not associated with reduced dosages of bDMARDs (OR 1.14, 95% CI 0.79–1.64), whereas ARBs users tended to use less bDMARDs (1.46, 95% CI 0.98–2.18, *p* = 0.06). Conclusion: In this study, the use of either ACEi or ARBs in RA patients had no impact on disease activity as measured by the DAS28-CRP. A trend towards lower bDMARD dosages was observed in ARBs users, but the significance of this finding is still unclear.

## 1. Introduction

Rheumatoid arthritis (RA) is a chronic inflammatory condition characterized by joint inflammation and several comorbidities. Notably, RA is associated with an increased risk of cardiovascular (CV) morbidity and mortality [1]. This increased CV risk is mainly attributed to accelerated atherosclerosis, which can rely on traditional risk factors (including smoking, obesity, hypertension, and dyslipidaemia), as well as non-traditional/RA-related factors [2]. From among the latter, systemic inflammation seems to be pivotal [3], but other factors, including a certain genetic background and impaired physical activity, might add to the increased CV risk in these patients [4]. Particularly noteworthy are population-based studies that have indicated an increased prevalence of CV events even prior to RA diagnosis, with a slightly higher prevalence of hypertension and coronary events [5]. In addition, traditional CV risk factors alone cannot account for the total CV risk seen in RA patients [2]. It is worth mentioning that all these factors are interacting with each other in a complex network, in which bidirectional and synergistic effects can occur, including inflammation [6]. 

The relationship between RA and CVD stresses that keeping disease activity as low as possible is also important from a CVD perspective. This suggests that anti-inflammatory therapy with the use of Disease Modifying Anti-Rheumatic Drugs (DMARDs) might favorably impact cardiovascular risk in RA [6,7]. Several classes of DMARDs are currently available to control RA joint inflammation, with csDMARDs and bDMARDs being the most used by far, and tsDMARDs being the newest on the market. A recent narrative review on the effect of DMARDs on cardiovascular endpoint in RA indicated that while all DMARDs may lower CV risk by reducing inflammation, some are likely to favor CVD [6]. More specifically, methotrexate and hydroxychloroquine may improve blood pressure, whereas leflunomide might be associated with higher blood pressure and the risk of incident hypertension in these patients [8]. In contrast to leflunomide, bDMARDs were not associated with higher blood pressure and/or incident hypertension [8].

Hypertension is an important risk factor for CV and its prevalence in RA patients is relatively high—between 40 and 45%, depending on the study [2]. Moreover, previous studies have indicated that hypertension is undertreated in RA patients [2,9]. This underlines the importance of containing traditional risk factors, such as hypertension in RA, in order to have a significantly favorable impact on CV risk in these patients. Angiotensin-converting enzyme inhibitors (ACEi) and angiotensin II receptor blockers (ARBs) are among the first-line anti-hypertensive drugs recommended by treatment guidelines worldwide [10]. As ACEi treatment blocks the conversion of angiotensin I to II—preventing the binding to both angiotensin type I (AT1) and II (AT2) receptors—it may be less preferable to ARBs, which decrease vasoconstriction by AT1-receptor binding while leaving the AT2 receptors to facilitate vasodilation.

To our knowledge, the effect of ACEi and ARBs on disease activity in larger groups of RA patients, measured with the disease activity score28 using C-reactive protein (DAS28-CRP), has not been investigated so far. Potential clinically relevant anti-inflammatory effects might also be accompanied by a reduced DMARD usage, as remission in RA is usually followed by medication tapering. Therefore, the present study primarily aims to investigate whether the use of ACEi and ARBs is associated with lower disease activity scores and/or less DMARDs use in patients with rheumatoid arthritis. In addition, the levels of CRP and kidney function is also investigated, as both are strongly associated with the risk of developing future CV events. 

## 2. Methods

### Study Design

The study is a retrospective, cross-sectional data study. Data for this study were obtained from the IRIS database. This database consists of clinical and medication data from all the outpatient visits of RA patients of the Sint Maartenskliniek. All patients gave their informed consent for the use of their data for scientific purposes, in accordance with the current laws and regulations. Given its retrospective character, the study was exempted from full ethical review by the Regional Board and was approved by the Local Institutional Review Board of the Sint Maartenskliniek, in accordance with the Dutch Legislation on this matter. Additional medication data were extracted from the pharmacy database of the Sint Maartenskliniek (RR-202-LOT). Patients visited our outpatient clinic between 1 May 2019 and 30 April 2020. The data corresponding to the last visit of every patient in the above-mentioned time interval (with a DAS28-CRP score) were extracted for the purpose of this study.

## 3. Participants

For the purpose of this study, we used retrospective data that were gathered from the IRIS database in the Sint Maartenskliniek. In short, this database comprises information from patients with various inflammatory rheumatic conditions, including RA, that were treated in our clinic in the past years. All adult (18 years of age or older) patients with an ACR/EULAR or clinical diagnosis of rheumatoid arthritis made by their rheumatologist were eligible. It was mandatory for patients to have undergone treatment by a rheumatologist in the Sint Maartenskliniek, and to have had at least one appointment at the outpatient clinic. A DAS28-CRP score was taken between 1 May 2019 and 30 April 2020. There were no other exclusion criteria. 

### 3.1. Outcomes of the Study

The primary outcome consisted of the differences between the last registered DAS28-CRP score of RA patients with and without ACEi or ARBs therapy. The secondary outcomes were set as the average dosage of the biological DMARD (bDMARD) and the conventional synthetic DMARD (csDMARD) expressed in DDD (Defined Daily Dose), at the same time point as the last registered DAS28-CRP. In addition, a sub-analysis of the DAS28-CRP scores was performed in the group of RA patients with concomitant hypertension. The DDD was based on the Anatomical Therapeutic Chemical classification system/Defined Daily Dose (ATC/DDD) methodology of the WHO [11]. The DDD was based on the treatment of rheumatoid arthritis. For rituximab, there was no DDD determined in the ATC index for rheumatoid arthritis. The DDD used for rituximab was based on average maintenance dosage in the Sint Maartenskliniek—1000 mg every 6 months. For the same reason, the DDD of hydroxychloroquine was set at 400 mg per day. 

### 3.2. Exposure to ACEi and ARBs

Data from the pharmacy database of the Sint Maartenskliniek and clinical data from the IRIS database were combined to select all ACEi and ARBs users with a DAS28-CRP score between May 2019 and May 2020. Control patients were randomly selected from the whole RA patient population at the Sint Maartenskliniek using the same IRIS database, given that they had a DAS28-CRP score in the above-mentioned time interval.

### 3.3. Measurements

The estimated glomerular filtration rate (eGFR) and C-reactive protein (CRP) were measured according to treatment protocol during the outpatient visits and were included if they were registered within 3 months before or after the measurement of the DAS28-CRP score. The last known weight and height were included. Medication data were collected during the whole inclusion period, as previously stated. Age and disease duration were measured on 1 May 2020. Comorbidities, anti-CCP antibodies, and the rheumatoid factor were included from the patient’s medical history. 

### 3.4. Statistical Analysis

Descriptive data were described either as a mean, with a standard deviation (parametric data), or as a median, with a 25th and 75th percentile (nonparametric data). For statistical comparisons, two-samples *t*-test were used for continuous variables, whereas proportion was tested with a Chi-square test. For the non-parametric data, a Wilcoxon rank-sum test was used. The DAS28-CRP scores, DMARD dosages, and the sub-analysis of patients with hypertension were studied using a multiple regression analysis. In order to reduce the risk of confounding, the following confounders were included in the regression analysis, based on evidence in existing literature: age, sex, disease duration, smoking, weight, oral steroid use, NSAIDs use, diabetes, and eGFR. The eGFR was included in the analysis as either impaired kidney function (eGFR under 60) or not. The rheumatoid factor and anti-CCP antibodies were combined in the analysis (as seropositive and seronegative RA, respectively) due to collinearity. 

From among all the RA patients visiting the outpatient clinic at the Sint Maartenskliniek during the study period, 584 ACEi and ARB users were identified and included in the study. A group of 552 control RA patients were randomly selected from the whole RA population at the Sint Maartenskliniek, which currently provides treatment to more than 4000 RA patients. In the linear regression, DAS28-CRP scores underwent log transformation for normal distribution. Disease duration also underwent transformation due to non-linearity; this transformation was $\frac{1}{\sqrt{Diseaseduration}}$). In order to differentiate between the individual effects of the ACEi (*n* = 335) and ARB (*n* = 249), both drug classes were analyzed separately. Due to the relatively low number of patients with specific ACEi and/or ARBs, we decided to perform the analysis for the whole class and not separately for each drug. The DDD of csDMARDs underwent log transformation in order to create normal distribution, and disease duration underwent log transformation due to non-linearity. Additionally, a logistic regression analysis of the DDD of the bDMARDs was performed due to their non-parametric and non-linear character. The logistic regression was performed with the standard dosage (DDD ≥ 1) or the reduced dosage (DDD < 1). A *p* value of <0.05 was considered statistically significant in all statistical tests. Statistical analysis was performed using Stata/IC version 13. All data relevant to the study were included in the manuscript. 

## 4. Results

The ACEi and ARB users were older, predominantly male, had a longer disease duration, frequently impaired kidney function, and had significantly more comorbidities, including diabetes, hypertension, and acute myocardial infarction (Table 1). For this reason, age, sex, disease duration, kidney function, diabetes, hypertension, and cardiovascular disease were used as possible confounders in the linear regression model. Due to a large number of missing values in the collected data, BMI, smoking, and alcohol consumption could not be used as confounders in the analysis.

### 4.1. Effect of ACEi and/or ARBs on Disease Activity

A multiple linear regression analysis was performed in order to compare the effect on the DAS28-CPR scores (Table 2). Neither the use of ACEi nor ARBs was significantly associated with disease activity. Similarly, no differences could be seen in the tender joint count, whereas the number of swollen joints tended to be lower in ACEi/ARBs users (Table 1). Female patients had a 10% higher DAS28-CRP score in comparison to their male counterparts (Female factor 1.10, *p* < 0.001). With regard to using NSAIDs, the use of steroids and hypertension were associated with higher DAS28-CRP scores as well (Table 2). Conversely, longer disease duration and csDMARDs use were associated with lower DAS28-CRP scores (Table 2). In a sub-analysis of 274 patients where the BMI was known, there was no significant association found between the BMI and DAS28-CRP of the ACEi/ARBs users and non-users, respectively (Table 2).

### 4.2. Effect of ACEi and/or ARBs Use on Medication Use 

We further investigated whether the use of ACEi/ARBs would lead to a reduction of DMARDs dosage, which is common practice when RA patients enter longer periods of remission or low disease activity (LDA). In other words, if the putative anti-inflammatory effect of ACEi/ARBs did not manifest in DAS28-CRP differences, it might lead to the tapering of DMARDs as a result of stable disease activity. Therefore, a lower DDD would reflect a longer period of remission or LDA. Consequently, we performed a multiple linear regression analysis to investigate the possible effect of ACEi /ARBs use on the DDD of csDMARDs and bDMARDs, respectively, and found no significant associations with the DDD of csDMARDs (Table 3). Using steroids, having seropositive RA, and disease duration had a statistically significant association with a higher DDD of csDMARDs, whereas impaired kidney function (factor 0.85, P-value < 0.005) was associated with a lower DDD of csDMARDs (Table 3).

While observing the DDD of bDMARDs, the use of ACEi still could not be significantly associated with a lower DDD of these drugs (Table 4). Nevertheless, we found a trend towards a lower bDMARDs use when the same analysis was performed on users of ARBs (Table 4). Other factors were found to influence the DDD of bDMARDs in our group, which include the use of NSAIDs, having seropositive RA, longer disease duration, and lower age (Table 4). The uncorrected means are the following: for the control group, 0.82 (95% CI 0.78–0.86); for the ACEi group, 0.77 (95% CI 0.72–0.82); and for the ARB group, 0.79 (95% CI 0.74–0.84). 

A sub-analysis performed on RA patients with hypertension (*N* = 357) yielded similar results to those found in the whole cohort. The effect of ACEi and ARBs on DAS28-CRP scores (factor 1.03, *p* = 0.64 and factor 1.06, *p* = 0.33, respectively), the DDD of csDMARDs (factor 0.88, *p* = 0.24 and factor 0.93, *p* = 0.48), and the DDD of bDMARDs (ACEi OR 1.27, *p* = 0.80; ARB OR 1.44, *p* = 0.33) were all not statistically significant. 

## 5. Discussion

In this retrospective cross-sectional study, we found no association between the use of ACEi or ARBs and lower disease activity in RA patients, as assessed by DAS28-CRP scores. We also found no significant association between the use of either ACEi or ARBs and lower dosages of csDMARDs or bDMARDs in these patients. Nevertheless, there seems to be a trend among users of ARBs towards a reduced dosage of bDMARDs. This might be due to the anti-inflammatory effects of some of the ARBs, as demonstrated in previous studies.

Our study is the first to assess the clinical effects of ACEi and ARBs in a large, real-life cohort of patients with RA. Previous studies investigating the effect of ACEi and ARBs in animal models of arthritis [12,13,14,15] have suggested a putative, anti-inflammatory effect of these drugs and proposed that they might decrease disease activity in RA patients. Small trials have also been performed in RA patients but yielded controversial results [16,17]. In our study, the use of ACEi and ARBs seemed to have no additional suppressive effect on disease activity, as measured by DAS28-CRP and on top of that elicited by the therapy with csDMARDs and bDMARDs, respectively. 

Some explanations could be provided to sustain our results. Firstly, the additional anti-inflammatory effect of ACEi and ARBs, if present, might be of lesser clinical impact than that of all DMARDs. Our hospital strictly follows the treat-to-target treatment strategy [18], according to which remission and/or low disease activity (LDA) become the main therapeutic goals. A higher DAS28 would result in more aggressive anti-rheumatic therapy with DMARDs, whereas a lower DAS28 would determine opposite actions. In this equation, the introduction of ACEi/ARBs is likely to be of little impact, so that the DAS28 would remain the same on the group level. Secondly, ACEi and ARBs may vary in terms of their anti-inflammatory potencies. That would mean that the stronger anti-inflammatory effects of some ACEi and ARBs might be neutralized by the rest when assessed together in a group and as a class. Differences in anti-inflammatory effects may be seen not only within the same class, but also between some ACEi and ARBs. Accordingly, Schieffer et al. found similar effects of enalapril and irbesartan on the MMP-9 levels, but only the therapy with irbesartan was followed by lower CRP and IL-6 in patients with coronary artery disease; no such effect was observed when enalapril was used [19]. This is in line with a previous study indicating that enalapril could not suppress IL-6 production from stimulated PBMCs, whereas losartan, another ARB drug, was able to do so [20]. Considering these facts, one may speculate that ARBs might have a stronger anti-inflammatory effect than ACEi. Nevertheless, we are uncertain whether this provides the main explanation for our results, as enalapril accounted for just a small part (8.7%) of the patients in the ACEi/ARBs group. 

**Figure 1:**
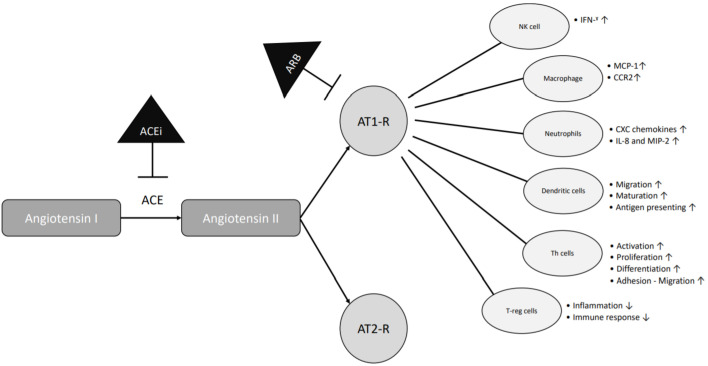
Anti-inflammatory mechanisms shared by ACE inhibitors and ARBs. ACE = angiotensin-converting enzyme; ACEi = angiotensin-converting enzyme inhibitor; AT1-R = angiotensin receptor type 1; AT2-R = angiotensin receptor type 2; ARB = angiotensin receptor type 1 inhibitor; NK = natural killer; Th = T helper cells; T-reg = T regulatory cells; IFN = interferon; MCP-1 = monocyte chemoattractant protein -1; CCR2 = C-C chemokine receptor 2; CXC = chemokines; IL-8 = interleukin-8; MIP-2 = macrophage inflammatory protein-2.

Interestingly, the stimulation of the AT1 receptor may elicit pro-inflammatory effects, including nuclear factor (NF)-kB activation, which leads to the production of pro-inflammatory cytokines, chemokines, and adhesion molecules by resident cells, thereby amplifying the inflammatory responses [21,22,23,24] (Figure 1). Even bone erosion is increased after upregulation of angiotensin II in mice [12]. Conversely, the blockade of the renin-angiotensin system (RAS) has been suggested to dampen inflammation and protect against arthritis. Murine studies indicate that ACEi and ARBs are as potent as methotrexate (MTX) or dexamethasone in reducing serum CRP, TNF-α, and IL-10 levels, while at the same time preventing cartilage erosions [13]. In addition, losartan was reported to reduce IFN gamma, IL-6, IL-17F, and IL-22 production from stimulated PBMCs from RA patients [20], whereas this was not seen when enalapril or valsartan were used, suggesting that anti-inflammatory properties might be drug-specific. Several studies have shown the beneficial effects of the use of ACEi and ARBs in animal models of induced arthritis [14,25,26,27]. Likewise, captopril has been shown to lower disease activity in RA [16,21,28], whereas an open label study using pentopril found no clinical improvement despite a decrease in CRP levels [17]. Nevertheless, the use of ACEi and ARBs was not associated with incident RA in a Dutch matched case-control study [29]. Taken together, these studies bring inconclusive evidence for a strong and beneficial anti-inflammatory effect of ACEi and ARBs in RA. If present, the anti-inflammatory effects might be drug specific. 

Another intriguing finding of our study was the trend towards a lower bDMARDs dosage in the group of RA patients using ARBs, but not in those using ACEi. This suggests that ARBs might be more potent in combating inflammation in RA than ACEi. ARBs may lead to decreased cytokine production or reduced leukocyte rolling and adhesion, as suggested by Silveira et al. in animal models [15]. In addition, the use of ARBs was followed by functional improvement and less pain in the affected joints. Using animal models of arthritis, Price et al. further suggested that ARBs, if dosed properly, could lead to reduced joint swelling [14]. Reducing the dose of bDMARDs after achieving remission or LDA is a common practice embedded in the RA treatment protocol of our clinic. Thus, a lower bDMARDs dosage might mirror lower cumulative disease activity and a longstanding remission. However, the association with more users of ARBs in this group of RA patients is, in our opinion, not strong enough to prove causality. Nevertheless, given the literature suggesting the anti-inflammatory potential of ARBs, it might be best to further investigate this hypothesis in larger studies, using designs that overcome the limitations of our present study.

The relatively large number of patients included in our study, by using the maximal convenience sample, strengthened the results obtained. Moreover, since no drastic exclusion criteria were used, we expect the data presented to closely mirror the situation in the daily clinical rheumatology practice. Despite these facts, there is room for improvement with respect to the results obtained in the present study and their interpretation. Some of these improvements might be related to the present design (retrospective, cross-sectional, choice of cohort, missing data, etc.). Others will still be difficult to achieve, as with the use of NSAIDs (over the counter drugs with an impact on DAS28 components) or the poor drug adherence observed in RA [30]. Finally, the results of the present study encourage further investigation of a possible link between ACEi/ARBs and RA, with a specific focus on whether effects apply to a whole class or only to some of the drugs within this class.

ACEi and ARBs are important in maintaining blood pressure within normal levels and in protecting against future CV events, which is normally increased in the RA population. Patients with hypertension and RA who use an ARB have a lower hazard ratio of 0.641 for the first acute myocardial infarction as compared to non-users [31]. Higher angiotensin II serum levels were observed in RA even before the diagnosis of RA was made, which could explain the higher incidence of hypertension among these individuals [5,32]. Side effects have a low incidence; these are lower for ARBs than for ACEi [33]. Corroborating with the results of our study, these data argue for the preferential use of ARBs in RA hypertensive patients as first-line anti-hypertensive drugs. Nevertheless, more evidence should be provided by future studies in order to strengthen this proposition, ideally in a prospective manner or in a randomized controlled trial. 

In conclusion, our study could not find any association between the use of ACEi and ARBs, and lower RA disease activity and lower DMARDs dosages, respectively. A trend of more ARBs users and a lower bDMARDs dosage was observed, although causation cannot be claimed based on the present study. Future studies should further verify whether ARBs and/or ACEi can in fact diminish disease activity in RA. If this is the case, these studies may confirm whether this effect is specific to a few drugs or if it could be generalized for the whole class. 

## Figures and Tables

**Table 1:** Demographic variables and patient characteristics.

	Exposed to ACEi or ARBs (*n* = 584)	Non-Exposed (*n* = 552)	*p* Value
Age, years ^a^	70.3 (10.0)	61.2 (14.6)	<0.01
Female, *n* (%)	382 (65.4)	395 (71.6)	0.03
Disease duration, years ^b^	12.8 (6.2–20.4)	8.0 (2.6–16.0)	<0.01
Positive rheumatoid factor (%)	63.7	65.0	0.66
Anti-CCP positive (%)	60.2	65.0	0.11
CRP ^b^	2 (1–7)	2 (1–6)	0.76
SJC ^b^	0 (0–1)	0 (0–1)	0.05
TJC ^b^	0 (0–1)	0 (0–1)	0.62
Kidney function (%)			
Impaired	21.4	7.1	<0.01
Steroid users (%)	17.3	16.1	0.63
NSAID users (%)	37.7	42.6	0.10
DMARD users (%)	85.8	77.4	<0.01
csDMARDs	72.4	62.9	<0.01
bDMARDs	38.9	33.0	0.04
DDD csDMARDs ^b^	0.9 (0.6–1.3)	1.0 (0.7–1.4)	<0.01
DDD bDMARDs ^b^	1.0 (0.5–1.0)	1.0 (0.6–1.0)	0.97
Medication (%)			
Enalapril	8.7		
Lisinopril	25.7		
Perindopril	13.9		
Losartan	18.2		
Irbesartan	7.2		
Other ACEi or ARB	26.3		
Comorbidities (%)			
Diabetes	17.3	5.4	<0.01
Hypertension	48.6	13.2	<0.01
Other cardiovascular diseases	38.2	16.7	<0.01
CVA	5.8	3.8	0.13
TIA	5.1	2.7	0.05
AMI	12.5	2.0	<0.01
AP	4.5	2.5	0.11

^a^ = mean (sd); ^b^ = median (25th and 75th percentile); ACEi = angiotensin-converting enzyme inhibitors; ARB = angiotensin receptor blockers; Anti-CCP = anti-cyclic citrullinated peptide; CPR = C-reactive protein; eGFR = estimated glomerular filtration rate; DMARD = disease-modifying anti-rheumatic drug; csDMARD = conventional synthetic DMARD; bDMARD = biological DMARD; DDD = defined daily dose; CVA = cerebrovascular accident; TIA = transient ischemic attack; AMI = acute myocardial infarct; AP = angina pectoris.

**Table 2:** Multiple linear regression of multiple confounders and the effect on the DAS28-CRP scores.

**F(14, 1121)**	=		9.64	
**Prob > F**	=		0.0000	
**R-squared**	=		0.1074	
DAS28-CRP *	**Factor**	**(95% Conf.Interval)**		***p* > t**
Intercept	0.45 ^#^	0.30–0.60		<0.001
Using ACEi	1.00	0.94–1.06		0.965
Using ARB	1.02	0.96–1.09		0.483
Female	1.10	1.05–1.15		<0.001
Diabetes	1.01	0.94–1.08		0.848
Impaired kidney function	1.06	0.99–1.14		0.071
Using NSAIDs	1.05	1.00–1.10		0.050
Using steroids	1.16	1.09–1.23		<0.001
Seropositive RA	0.98	0.93–1.03		0.343
CVD	1.01	0.96–1.07		0.643
Hypertension	1.06	1.00–1.12		0.036
BMI ^##^	1.01	1.00–1.02		0.070
Disease duration *	0.57	0.50–0.66		<0.001
Age	1.00	1.00–1.00		0.076
Using csDMARDs	0.93	0.87–0.98		0.003
Using bDMARDs	1.01	0.96–1.06		0.776

* = DAS28-CRP transformed in ln(DAS28-CRP) due to normality, and disease duration transformed in (disease duration)^−0.5 due to linearity; ^#^ = intercept is no factor, B_0_; ^##^ = analysis performed in smaller group of 274 patients; ACEi = angiotensin-converting enzyme inhibitors; ARB = angiotensin receptor blockers; NSAIDs = non-steroidal anti-inflammatory drugs; CVD = cardiovascular disease; DMARD = disease-modifying anti-rheumatic drug; csDMARD = conventional synthetic DMARD; bDMARD = biological DMARD.

**Table 3:** Multiple linear regression of multiple confounders on the defined daily dose of csDMARDs.

**F (12, 757)**	=		11.10	
**Prob > F**	=		0.0000	
**R-squared**	=		0.1496	
DDD csDMARDs *	**Factor**	**(95% Conf. Interval)**		***p* > t**
Intercept	0.43 ^#^	0.21–0.65		<0.001
Using ACEi	0.97	0.89–1.07		0.547
Using ARB	0.99	0.90–1.10		0.914
Female	0.95	0.88–1.02		0.157
Diabetes	1.08	0.96–1.21		0.201
Impaired kidney function	0.85	0.76–0.94		0.002
Using NSAIDs	1.02	0.94–1.10		0.658
Using steroids	1.11	1.01–1.24		0.032
Seropositive RA	1.10	1.01–1.19		0.024
CVD	0.99	0.91–1.07		0.750
Hypertension	1.04	0.95–1.13		0.396
Disease duration *	2.34	2.27–2.42		<0.001
Age	1.00	0.99–1.00		0.070

* DDD csDMARDs transformed in ln(DDD csDMARDs) due to normality, and disease duration transformed in ln(disease duration) due to linearity; ^#^ = intercept is no factor, B_0_; ACEi = angiotensin-converting enzyme inhibitors; ARB = angiotensin receptor blockers; NSAIDs = non-steroidal anti-inflammatory drugs; CVD = cardiovascular disease; DMARD = disease-modifying anti-rheumatic drug; csDMARD = conventional synthetic DMARD; bDMARD = biological DMARD.

**Table 4:** Logistical regression of multiple confounders on the defined daily dose of bDMARDs, standard dosage.

**LR chi^2^(12)**	=		48.85	
**Prob > chi^2^**	=		0.0000	
**Pseudo R^2^**	=		0.0395	
**bDMARD**	**Odds Ratio**	**(95% Conf. Interval)**		***p* > z**
Intercept	0.23	0.10–0.56		0.001
Using ACEi	1.14	0.79–1.64		0.477
Using ARB	1.46	0.98–2.18		0.061
Female	0.91	0.66–1.23		0.531
Diabetes	0.94	0.59–1.50		0.790
Impaired kidney function	0.96	0.62–1.48		0.854
Using NSAIDs	1.39	1.04–1.86		0.028
Using steroids	1.02	0.70–1.47		0.929
Seropositive RA	2.09	1.46–2.98		<0.001
CVD	1.21	0.86–1.70		0.274
Hypertension	0.90	0.64–1.27		0.550
Disease duration	1.03	1.01–1.04		<0.001
Age	0.99	0.97–1.00		0.044

ACEi = angiotensin-converting enzyme inhibitors; ARB = angiotensin receptor blockers; NSAIDs = non-steroidal anti-inflammatory drugs; CVD = cardiovascular disease; DMARD = disease-modifying anti-rheumatic drug; csDMARD = conventional synthetic DMARD; bDMARD = biological DMARD.

## Data Availability

All data relevant to the study have been included in the manuscript.

## Abbreviations

RA	rheumatoid arthritis
CVD	cardiovascular disease
CV	cardiovascular
ACE	angiotensin-converting enzyme
ARB	angiotensin II receptor blockers
RAS	renin-angiotensin system
DMARDs	disease-modifying antirheumatic drugs
NSAIDs	non-steroidal anti-inflammatory drugs
ACR/EULAR	American College of Rheumatology/European League Against Rheumatism
DAS28-CRP	Disease Activity Score-28
eGFR	estimated glomerular filtration rate
CRP	C-reactive protein
bDMARD	biological disease-modifying antirheumatic drugs
csDMARD	conventional synthetic disease-modifying antirheumatic drugs
DDD	defined daily dose
ATC/DDD	Anatomical Therapeutic Chemical classification system/ Defined Daily Dose
Anti CCP	anti-cyclic citrullinated peptide
CVA	cerebrovascular accident
TIA	transient ischemic attack
AMI	acute myocardial infarct
AP	angina pectoris
